# Risperidone-Induced Agranulocytosis in a Young Patient With Schizophrenia: A Case Report

**DOI:** 10.1192/j.eurpsy.2026.11767

**Published:** 2026-07-31

**Authors:** O. Seyar, L. Azizi, Z. Bencharfa, F. El Omari

**Affiliations:** 1 psychiatry; 2 Arrazi hospital, Salé, Morocco

## Abstract

**Introduction:**

Risperidone is a widely prescribed atypical antipsychotic. Hematological adverse effects, although well documented with clozapine, are rarely associated with risperidone. We present a case of recurrent risperidone-induced agranulocytosis in a young patient with schizophrenia.

**Objectives:**

To describe a rare case of risperidone-induced agranulocytosis in a young patient with schizophrenia, to explore its possible immuno-allergic mechanism, and to highlight the importance of hematological monitoring during antipsychotic treatment.

**Methods:**

A 23-year-old Moroccan male with a 5-year history of schizophrenia and no relevant medical history was treated with risperidone after inadequate response to olanzapine. The dose was increased from 2 to 6 mg within four days, with good initial efficacy. Clinical monitoring and serial complete blood counts (CBC) were performed throughout treatment and rechallenge.

**Results:**

Six weeks after risperidone initiation, progressive leukopenia and neutropenia were observed, with neutrophils falling to 750/μL after two months, leading to drug discontinuation. Chlorpromazine was also stopped, and aripiprazole (15 mg/day) was introduced, resulting in hematological recovery but poor clinical response. Risperidone rechallenge under daily CBC monitoring caused a more rapid recurrence of neutropenia within one week, supporting an immune-allergic mechanism. Alternative explanations such as ethnic neutropenia, margination, or concomitant medications (chlorpromazine, hydroxyzine) were excluded. A literature review identified 27 similar cases of risperidone- or paliperidone-induced neutropenia. This case illustrates the rare but serious risk of risperidone-induced agranulocytosis. The accelerated recurrence upon rechallenge strongly suggests hypersensitivity rather than dose-related toxicity. Although considered rare by pharmacovigilance reports, this adverse effect may lead to severe infectious complications if unrecognized.

**Conclusions:**

Baseline and follow-up CBC monitoring should be performed after risperidone initiation, even though hematological toxicity is uncommon. The use of long-acting risperidone formulations should be delayed until tolerance to the oral form is established.

**Disclosure of Interest:**

None Declared

